# A global, historical database of tuna, billfish, and saury larval distributions

**DOI:** 10.1038/s41597-022-01528-7

**Published:** 2022-07-19

**Authors:** Kristine Camille V. Buenafe, Jason D. Everett, Daniel C. Dunn, James Mercer, Iain M. Suthers, Hayden T. Schilling, Charles Hinchliffe, Alvise Dabalà, Anthony J. Richardson

**Affiliations:** 1 School of Earth and Environmental Sciences, The University of Queensland, St Lucia, QLD 4067 Australia; 2 School of Mathematics and Physics, The University of Queensland, St Lucia, QLD 4067 Australia; 3 Commonwealth Scientific and Industrial Research Organization (CSIRO) Oceans and Atmosphere, Queensland Biosciences Precinct (QBP), St Lucia, QLD 4067 Australia; 4 Centre for Marine Science and Innovation (CMSI), The University of New South Wales, Sydney, NSW 2052 Australia; 5 Sydney Institute of Marine Science, Mosman, NSW 2088 Australia; 6 Systems Ecology and Resource Management, Department of Organism Biology, Faculté des Sciences, Université Libre de Bruxelles - ULB, Avenue F.D. Roosevelt 50, CPi 264/1, 1050 Brussels, Belgium; 7 Ecology and Biodiversity, Laboratory of Plant Biology and Nature Management, Biology Department, Vrije Universiteit Brussel - VUB, Pleinlaan 2, VUB-APNA-WE, 1050 Brussels, Belgium

**Keywords:** Marine biology, Conservation biology

## Abstract

Knowing the distribution of fish larvae can inform fisheries science and resource management in several ways, by: 1) providing information on spawning areas; 2) identifying key areas to manage and conserve; and 3) helping to understand how fish populations are affected by anthropogenic pressures, such as overfishing and climate change. With the expansion of industrial fishing activity after 1945, there was increased sampling of fish larvae to help better understand variation in fish stocks. However, large-scale larval records are rare and often unavailable. Here we digitize data from Nishikawa *et al*. (1985), which were collected from 1956–1981 and are near-global (50°N–50°S), seasonal distribution maps of fish larvae of 18 mainly commercial pelagic taxa of the families Scombridae, Xiphiidae, Istiophoridae, Scombrolabracidae, and Scomberesocidae. Data were collected from the Pacific, Atlantic, and Indian Oceans. We present four seasonal 1° × 1° resolution maps per taxa representing larval abundance per grid cell and highlight some of the main patterns. Data are made available as delimited text, raster, and vector files.

## Abstract (summary)

**Table:** 

Measurement(s)	larva abundance
Technology Type(s)	tows
Factor Type(s)	location of tows
Sample Characteristic - Organism	fish taxa
Sample Characteristic - Environment	open-ocean
Sample Characteristic - Location	near-global (Atlantic, Pacific, and Indian Oceans)

## Background & Summary

Fisheries help ensure global food security, with over 80 million tons of marine resources harvested annually, representing 17% of animal protein intake globally^1^. Much of the growth in the fisheries industry was caused by the expansion of longline fisheries after the end of World War II, particularly driven by the growing Japanese tuna market^2^. Accompanying this expansion was a growing number of process and field studies to help understand and manage fish populations. Most of the focus was on adult fish^3,4^, but because the spawning areas of most species were unknown—or known only for specific areas^5–8^—there came an increase in surveys of fish larvae. The largest of these post-war surveys (1956–1981) was Nishikawa *et al*. (1985). It contains near-global, historical data on larval distributions of fish species at 1° spatial resolution. Aspects of this dataset have been used in fisheries reports^9^ and in an analysis of seven tuna species on 5° grid squares^10^, but the data are not publicly available.

The Nishikawa larval abundance data should be valuable in at least three main research areas. The first is identifying potential key spawning areas and their environmental drivers. Spawning habitats can differ from the broad distribution of a fishery, as many species migrate to spawn in specific areas to optimize egg and larval survival^11^. These spawning habitats can be identified using raw larval abundance data. Alternatively, the same raw data could be combined with environmental data to create habitat suitability models^10,12–14^. Such models have the advantage of providing larval abundance estimates in areas with no sampling (i.e., they can fill in the spatial gaps in the raw data). Habitat suitability models can also provide insights into the potential environmental drivers of fish spawning.

The second area that the Nishikawa data could be used is in marine spatial planning^15–17^. Areas of overlap in spawning hotspots of many fish species could be focal areas for marine protected area networks in the high seas. Further, the Nishikawa data could be used to inform the establishment of other effective area-based conservation measures such as fisheries closures^18–21^. These closures restrict fishing effort around spawning aggregations that are vulnerable to fishing^16,22,23^, allowing overexploited fish stocks to recover^16,19,24^. Spatially and temporally resolved larval fish data can also provide evidence to justify and inform the establishment of seasonal closures^25,26^. Spawning areas separated in time and space can also be used to potentially identify valuable fish stocks^27^.

The third major research area in which the Nishikawa data could be used is to investigate changes in fish populations in response to anthropogenic pressures, such as overfishing and climate change^28–31^. Historical larval distributions could be compared with more recent data, highlighting spawning areas that have remained unchanged, those that have disappeared, and those that have newly emerged. Such a comparison could help identify potential causes of any changes in the spawning distribution of species. Moreover, by combining historical larval abundance data with environmental parameters, it is possible to project impacts of climate change on the spawning areas, or spawning phenology^29,32^ of future fish populations^28,31,33–35^.

Here, we digitize charts from Nishikawa *et al*. (1985), containing near-global, historical data on larval distributions in 18 fish taxa. Original data were in seasonal, global charts of 1° × 1° resolution spanning 25 years (1956–1981). Sampling was biased towards Western Pacific regions, primarily because the plankton surveys were carried out by Japanese government institutions surveying tuna longline grounds^36^. The Nishikawa dataset is a global treasure that is a valuable baseline of spawning habitats for large pelagic fish during the mid-20th century in the Anthropocene. We hope that making what is probably the largest near-global larval dataset publicly available will encourage its extensive future use in novel ways.

## Methods

### Description of dataset

The Nishikawa *et al*. (1985) dataset contains fish larval data collected between 50°N–50°S seasonally from 1956–1981 in the Pacific, Indian, and Atlantic Oceans. A total of 63,017 tows were recorded. Data were collected by different organizations and in a range of different ways, but these data are not available for each tow. Thus, we only summarize some of the major differences in methodology described in Nishikawa *et al*. (1985).

Tows were conducted by two groups of vessels—larger research vessels and smaller local government vessels. Each vessel type used different sizes of conical larvae sampling nets. Research vessels used a larger net of 2.0 m diameter and 6.0 m length, with a 1.7 mm mesh in front that narrowed to a 0.5 mm mesh at the cod end. Local government vessels used a smaller net of 1.4 m diameter and 4.0 m length, with similar mesh sizes compared to the larger net used by research vessels. In terms of depth, research vessels did surface and subsurface tows, whereas local government vessels did surface tows only. Sub-surface tow depths rarely exceeded 50 m and were usually 20–30 m deep. Tows by research vessels were consistently done during the day, whereas tows by government vessels were done during the night until 1969. Then, in 1970 daytime sampling was introduced except for surveys in the Western Equatorial Pacific.

Because different tow methods were used, seasonal larval abundance per taxon was standardized to catch per unit effort (CPUE)^37^ or the number of larvae per 1,000 m^3^ water strained. We present data for the 18 taxa recorded in Nishikawa *et al*. (1985) (Table 1; note that this table also summarizes the species in each of the 18 taxa). They identified fish larvae morphologically, making it difficult to distinguish some specimens and groups to the species level^36^. Moreover, the species in taxa groups were not always specified. It was clear from Nishikawa *et al*. (1985) that Frigate tuna (*Auxis* spp.) consists of *A. thazard* and *A. rochei*^36,38^, and little tuna group (*Euthynnus* spp.) comprised three endemic species— *E. affinis*, *E. lineatus*, and *E. alletteratus*^36,38^. Species in the Bonitos group (*Sarda* spp.) were not specifically listed, but are assumed to be *S. orientalis, S. australis, S. chiliensis*, and *S. sarda*^36,38,39^. The sauries group (Family: Scomberesocidae) most likely consisted of the Pacific saury (*Cololabis saira*), Eastern South Pacific saury (*C. adocetus*), and saury pike (*Scomberesox saurus*)^36^. Finally, a few species were grouped in Nishikawa *et al*. (1985). For example, larval distributions have been grouped together for: (1) blue marlin (*Makaira mazara*) and Atlantic blue marlin (*M. nigricans*); (2) striped marlin (*Tetrapturus audax*) and white marlin (*Tetrapturus albides*); and (3) shortbill spearfish (*Tetrapturus angustirostris*) and longbill spearfish (*Tetrapturus pfluegeri*). Bluefin tuna distributions comprise both *Thunnus thynnus* (Atlantic and Mediterranean) and *Thunnus orientalis* (Pacific). The remaining distributions are for single species, consistent with what was reported in Nishikawa *et al*. (1985).

**Table 1:** Taxa from the families Scombridae, Xiphiidae, Scombrolabracidae, Scomberesocidae, and Istiophoridae included in the dataset.

Taxa	Common names	Taxa names reported in Nishikawa *et al*. (1985)	Updated taxa names and/or possible species (with distributions^39^) for taxa reported in Nishikawa *et al*. (1985)	Family
1	Yellowfin tuna	*Thunnus albacares* B.	—	Scombridae
2	Albacore	*Thunnus alalunga* B.	—	Scombridae
3	Skipjack tuna	*Katsuwonus pelamis* L.	—	Scombridae
4	Bluefin tuna	*Thunnus thynnus* L.	*Thunnus thynnus* L. (Atlantic Ocean, Mediterranean Sea)	Scombridae
			*T. orientalis* (Pacific Ocean)	
5	Southern bluefin tuna	*Thunnus maccoyii* C.	—	Scombridae
6	Bigeye tuna	*Thunnus obesus* L.	*Thunnus obesus* L. (Atlantic, Indian, and Pacific Oceans)	Scombridae
			*T. atlanticus* L. (Western Atlantic Ocean)	
7	Frigate tuna	*Auxis* spp.	*Auxis rochei* R. (Atlantic, Indian, and Pacific Oceans)	Scombridae
			*A. thazard* L. (Atlantic, Indian, and Pacific Oceans)	
8	Little tuna	*Euthynnus* spp.	*Euthynnus affinis* C. (Indo-Pacific Region)	Scombridae
			*E. alletteratus* R. (Atlantic Ocean)	
			*E. lineatus* K. (Eastern Pacific Ocean)	
9	Bonitos	*Sarda* spp.	*Sarda australis* M. (Southwest Pacific Ocean and Tasman Sea)	Scombridae
			*S. chiliensis* (Eastern Pacific Ocean)	
			*S. orientalis* (Indo-Pacific Region)	
			*S. sarda* B. (Atlantic Ocean)	
10	Slender tuna	*Allothunnus fallai* S.	—	Scombridae
11	Swordfish	*Xiphias gladius* L.	—	Xiphiidae
12	Longfin escolar	*Scombrolabrax heterolepis* R.	—	Scombrolabracidae
13	Sauries	—	*Cololabis saira* (North Pacific Ocean)	Scomberosocidae
			*C. adoceta* (Central and Eastern Pacific)	
			*Scomberesox saurus* (Atlantic Ocean and Mediterranean Sea)	
14	Black marlin	*Makaira indica* C.	*Istiompax indica* C. (Indo-Pacific Region)	Istiophoridae
15	Sailfish	*Istiophorus platypterus* S.	—	Istiophoridae
16	Blue marlin	*Makaira mazara* J.	—	Istiophoridae
	Atlantic blue marlin	*Makaira nigricans* L.	—	
17	Striped marlin	*Tetrapturus audax* P.	*Kajikia audax* P. (Indo-Pacific Region)	Istiophoridae
	White marlin	*Tetrapturus albidus* P.	*Kajikia albida* P. (Atlantic Ocean)	
18	Shortbill spearfish	*Tetrapturus angustirostris* T.	—	Istiophoridae
	Longbill spearfish	*Tetrapturus pfluegeri* R. & d.S.	—	

Common names and taxon names are consistent with the original charts from Nishikawa *et al*. (1985). When applicable, we provided updated taxa names and the possible species that compose the larger taxa (e.g., genera) reported in the Nishikawa dataset^38,39^.

### Digitization

**Fig. 1:**
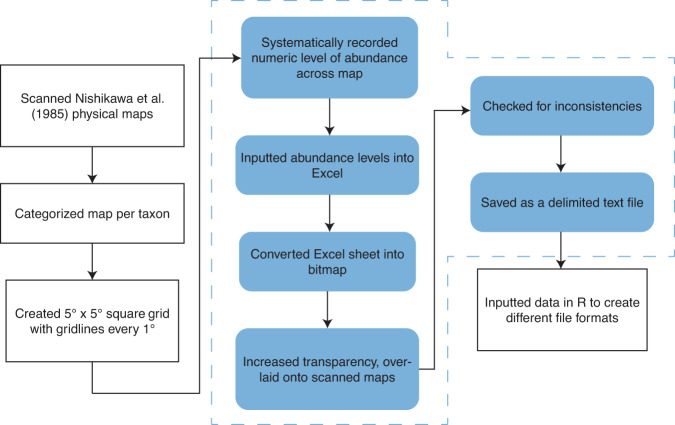
Summary of the digitization process. A flowchart of the process, with blue boxes repeated for all seasonal maps of the 18 taxa and towing effort.

The digitization process is summarized in Fig. 1. Original charts were scanned at 600 dpi. A 5° × 5° square grid, with gridlines every 1°, was overlaid on the scanned image of each chart. We first created template maps for each season by systematically moving the square grid from top-to-bottom, left-to-right of a seasonal chart, and repeating this for all four seasons. Since sampling areas per season were the same across all taxa, the templates were then used for digitizing all taxa larval charts.

The square grid was then moved systematically from top-to-bottom, left-to-right of each scanned chart. Categories of CPUE, represented by shapes on the scanned chart, were recorded as numeric levels (0–4) on a spreadsheet. This was done for the seasonal maps of 18 taxa, yielding a total of 72 digitized maps. Seasonal maps of tow effort (number of larval tows and volume of water strained) were digitized similarly. To validate the digitized maps, we saved the spreadsheets into semi-transparent bitmap formats, overlaid them on the scans of the charts, checked for any inconsistencies, and then updated the files if needed. Then, spreadsheets were converted to delimited text files (comma saved value files or.csv) and loaded into R^40^.

## Data Records

All data and code are found in a Zenodo Repository^41^. Digitized taxon maps are available as delimited text, raster^42^, and vector^43^ files. The files have the following headings: (1) Species; (2) Season; (3) Longitude; (4) Latitude; (5) Abundance; (6) FAO CWP Code^44^; and (7) FAO Major Fishing Areas^45^. Seasons were represented as month ranges: (1) January-March; (2) April-June; (3) July-September; and (4) October-December, as in Nishikawa *et al*. (1985). Abundance refers to the categorical numeric levels of CPUE, which represents different numerical values for different species (see Table 2).

**Table 2:** Catch per unit effort (CPUE) ranges for each category.

Common name	CPUE ranges (number of larvae·1000m^−3^)				
	0	1	2	3	4
Skipjack tuna	No catch	0.0–0.5	0.5–1.0	1.0–5.0	>5.0
Blue marlin and Atlantic blue marlin	No catch	0.0–0.5	0.5–1.0	1.0–3.0	>3.0
Yellowfin tuna	No catch	0.0–0.5	0.5–1.0	1.0–5.0	>5.0
Albacore	No catch	0.0–0.5	0.5–1.0	1.0–3.0	>3.0
Shortbill spearfish and longbill spearfish	No catch	0.0–0.5	0.5–1.0	1.0–3.0	>3.0
Frigate tuna	No catch	0.0–1.0	1.0–5.0	5.0–10.0	>10.0
Bigeye tuna	No catch	0.0–0.5	0.5–1.0	1.0–3.0	>3.0
Swordfish	No catch	0.0 – 0.5	0.5–1.0	1.0–3.0	>3.0
Striped marlin and white marlin	No catch	0.0–0.5	0.5–1.0	1.0–3.0	>3.0
Sauries	No catch	0.0–0.5	0.5–1.0	1.0–3.0	>3.0
Sailfish	No catch	0.0 – 0.5	0.5–1.0	1.0–3.0	>3.0
Longfin escolar	No catch	0.0 – 0.5	0.5–1.0	1.0–3.0	>3.0
Bluefin tuna	No catch	0.0 – 1.0	1.0–5.0	5.0–10.0	>10.0
Little tuna	No catch	0.0–0.5	0.5–1.0	1.0–3.0	>3.0
Southern bluefin tuna	No catch	0.0–0.5	0.5–1.0	1.0–5.0	>5.0
Slender tuna	No catch	0.0–0.5	0.5–1.0	1.0–3.0	>3.0
Bonitos	No catch	0.0–0.5	0.5–1.0	1.0–3.0	>3.0
Black marlin	No catch	0.0–0.5	0.5–1.0	1.0–3.0	>3.0

We also provide the digitized data for seasonal effort in delimited text file, raster^42^, and vector^43^ formats. The files have the following headings: (1) Category; (2) Season; (3) Longitude; (4) Latitude; (5) Effort; (6) FAO CWP Code^44^; and (7) FAO Major Fishing Areas^45^. Towing effort was expressed as either volume of water strained (Category: “Volume”) or number of net tows (Category: “Tows”). “Effort” values for the “volume” category refers to the ranges of water volume strained: 0 = < 5 × 10^3^ m^3^; 1 = 5.0–20.0 × 10^3^ m^3^; 2 = 20.0–30.0 × 10^3^ m^3^; 3 = 30.0–50.0 × 10^3^ m^3^; and 4 = ≥ 50.0 × 10^3^ m^3^. “Effort” values for the “tows” category refers to the ranges of number of tows: 0 = 1 tow; 1 = 2–5 tows; 2 = 6–15 tows; 3 = 16–30 tows; and 4 = ≥ 31 tows. Abundance and effort values are the seasonal averages of over 25 years of data collection.

**Fig. 2:**
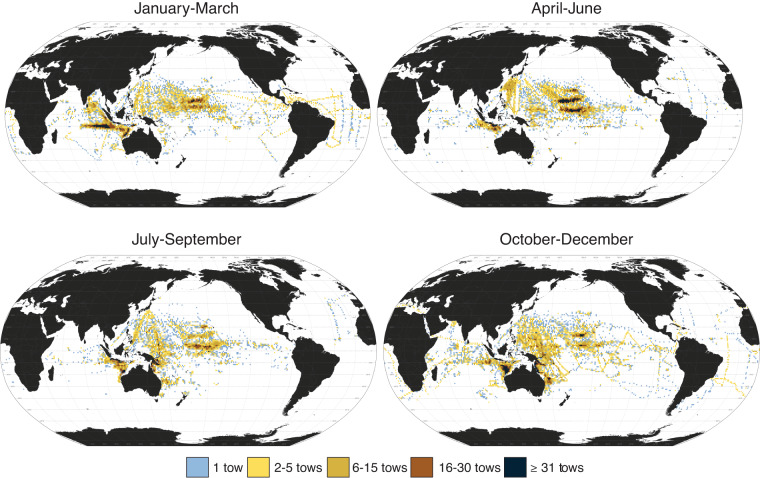
Seasonal towing effort map. Seasonal 1° × 1° maps of towing effort in terms of number of tows, with seasons (1956–1981) represented in ranges of months: (1) January-March; (2) April-June; (3) July-September; and (4) October-December.

The area sampled varied across seasons: (1) January-March (3,570 1° × 1° sampling areas); (2) April-June (2,854 1° × 1° sampling areas); (3) July-September (2,806 1° × 1° sampling areas); and (4) October-December (4,405 1° × 1° sampling areas). This seasonal sampling effort was consistent for all taxa. To aid interpretation, we provide maps of the seasonal effort: the number of tows (Fig. 2) and total volume of water filtered (Supplementary Figure S1). Towing effort is spatially and seasonally variable. There is higher confidence in the samples collected in areas of both higher number of tows (Fig. 2) and higher volume of water strained (Supplementary Figure S1). Surveys for all seasons were mostly concentrated in the Pacific Ocean, particularly the Western Pacific Ocean. In October-December there are more sampling areas in the Indian Ocean and Atlantic Ocean than any other season.

### Taxon maps

**Fig. 3:**
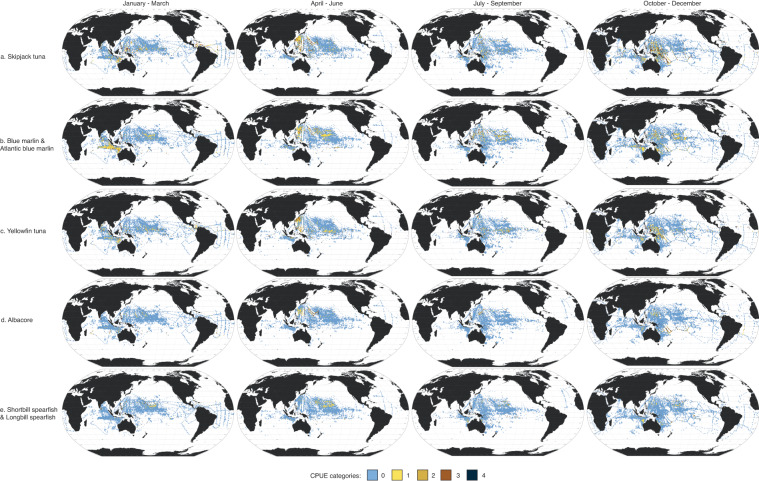

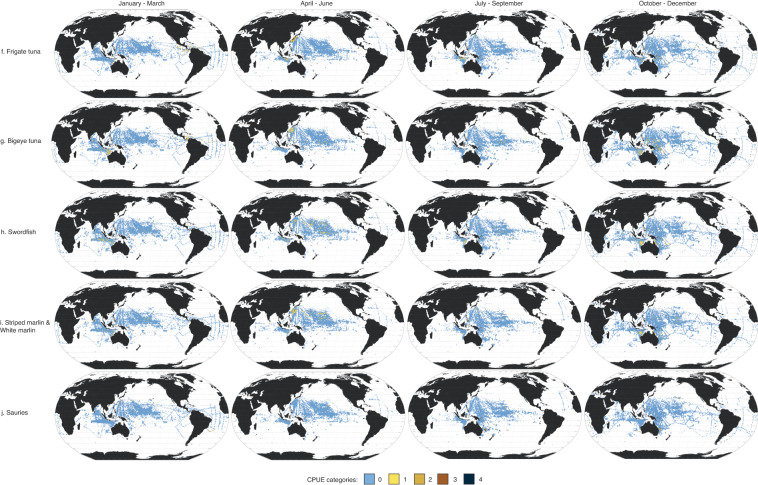

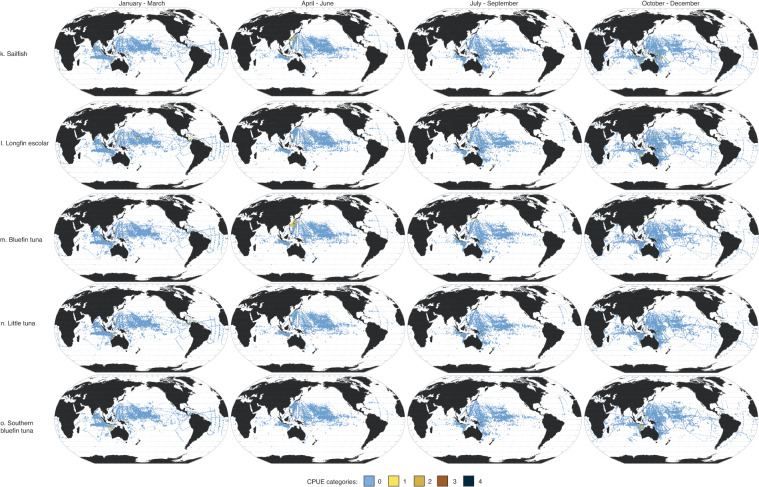

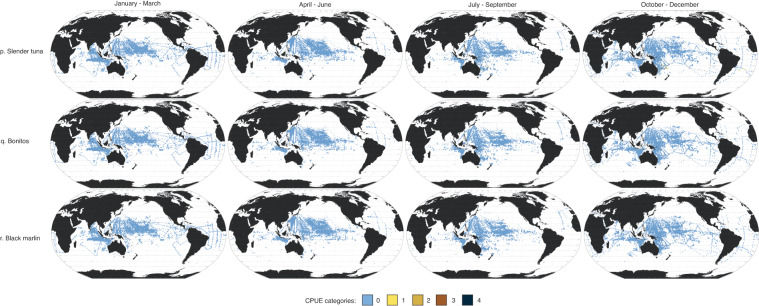
Seasonal larval distribution maps. Seasonal 1° × 1° maps of 18 taxa (**a**–**r**), with seasons (1956–1981) represented in ranges of months: (1) January-March; (2) April-June; (3) July-September; and (4) October-December. Catch per unit effort (CPUE) categories (0-4) for each species represent different CPUE ranges (expressed in number of larvae × 1000m^-3^; see Table 2).

Here, we present data in four seasonal maps for the 18 taxa using the Robinson projection (Fig. 3). Taxa are arranged in descending order of absolute abundance (i.e., sum of abundance per taxon across all seasons). The most abundant taxon was skipjack tuna (*Katsuwonus pelamis* L.), then blue marlin (*Makaira mazara*) and Atlantic blue marlin (*M. nigricans*). Three of the five most abundant taxa come from the Scombridae family, which can be difficult to identify to the species level. Of the thousands of samples collected per season, most had no fish larvae present. Most of the positive samples (i.e., sampling areas where larvae were recorded) were in the tropical (25°N–25°S) Pacific Ocean.

**Fig. 4:**
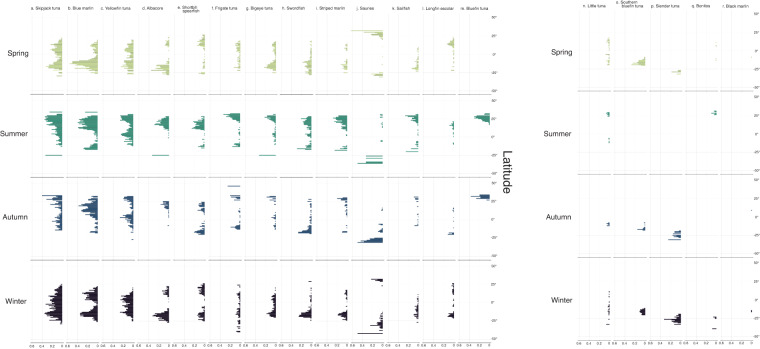
Seasonality of spawning hotspots. (**a**–**r**) Proportion of positive samples across latitudes (50°N–50°S) and seasons (1956–1981) for all 18 taxa.

To highlight the seasonality of potential spawning hotspots, we calculated the proportions of positive samples for each degree latitude from 50°N to 50°S for each of the 18 taxa (Fig. 4). This was calculated by counting the number of 1° × 1° sampling areas where a particular larva taxon is recorded and dividing it by the number of sampling areas in that latitude. Seasonality in potential spawning hotspots for taxa can be seen where the bar plot shifts or changes with season. For example, skipjack tuna larvae are present all-year round, having two distinct peaks around the subtropical latitudes in January to March, but widening in latitudinal range in April to September, and forming the subtropical peaks again in October to December (Fig. 4A). There are also taxa that show no seasonality, showing subtropical peaks across all seasons, like the yellowfin tuna (*Thunnus albacares*) (Fig. 4C), albacore (*T. alalunga*) (Fig. 4D), and shortbill spearfish (*Tetrapturus angustirostris*) (Fig. 4E). Some taxa were restricted both spatially and seasonally. For example, bluefin tuna larvae (*Thunnus thynnus* and *T. orientalis*) were only sampled from April to September, around 25°N (Fig. 4M). The confidence in these spawning hotspots could be considered by assessing the towing effort in each grid square seasonally (Fig. 2).

## Technical Validation

**Fig. 5:**
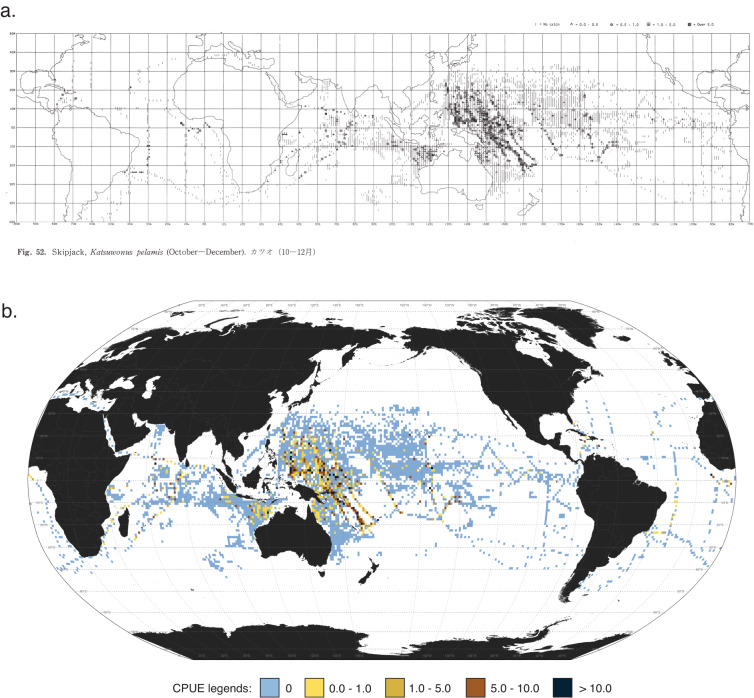
Side-by-side of seasonal map of skipjack tuna for October-December: (**a**) the original chart from Nishikawa *et al*. (1985); and (**b**) the digitized map.

Testing the validity and precision of the digitized maps could be done by comparing them with the data in the original charts. Here we provide an example of the digitized map and an original chart from Nishikawa *et al*. (1985) side-by-side (Fig. 5). The seasonal maps shown in this paper can be replicated using the scripts provided^41^. Seasonal maps could be overlaid on the scanned original charts. By increasing the transparency of either the map or the chart, each 1° × 1° data point should be counterchecked and verified in a systematic way from top-to-bottom and left-to-right of the entire chart. This should be repeated across the seasonal maps of the 18 taxa as well as the maps reporting the towing effort.

## Usage Notes

Original charts are found in Nishikawa *et al*. (1985). Digitized data in all formats (delimited text, vector, and raster files) are available online^41^. Larval distribution maps can be replicated by running the provided scripts. The delimited text file (.csv) shows latitudes and longitudes of the centroid of each 1° × 1° grid cell. The raster and vector files show data in 1° × 1° grid format. Vector files are generated per taxon per season and are saved as sf^43^ objects in R (.rds) with the Robinson projection. We have also provided a way to create unprojected vector files (with degree coordinates in longitude and latitude). We intersected the vector files with FAO’s Coordinating Working Party on Fishery Statistics (CWP) 1° × 1° areal grid system^44^ and major fishing areas^45^ to make the digitized data easier to use for fisheries statistical purposes. Raster files per taxon per season were saved as unprojected GeoTIFF (.tif) files, but the code to project these files to Robinson projection is also provided.

Some of the taxon maps are not specified to the species level. It is also specified and acknowledged in the Nishikawa dataset that some larvae are difficult to distinguish at the species level. For example, the bigeye tuna larvae closely resemble the Atlantic blackfin tuna larvae (*Thunnus atlanticus* L.)^46^ and the yellowfin tuna larvae (*T. albacares*), which means that the species maps provided may already include the distributions of the Atlantic blackfin tuna. There is also some difficultly differentiating sailfish (*Istiophorus platypterus*), white marlin (*Kajikia albida*), and blue marlin larvae (*Makaira mazara*)^47^. Hence, we recommend caution when interpreting these distribution maps.

### Supplementary information

Supplementary Figure S1

## Data Availability

All scripts are published at Zenodo under the identifier: 10.5281/zenodo.6592148/. To ensure that the code runs smoothly, use the updated versions of R and all Comprehensive R Archive Network (CRAN) packages declared in the repository. We used R version 4.0.3^40^.
