# Author Correction: A global, historical database of tuna, billfish, and saury larval distributions

**DOI:** 10.1038/s41597-023-02860-2

**Published:** 2024-01-04

**Authors:** Kristine Camille V. Buenafe, Jason D. Everett, Daniel C. Dunn, James Mercer, Iain M. Suthers, Hayden T. Schilling, Charles Hinchliffe, Alvise Dabalà, Anthony J. Richardson

**Affiliations:** 1 School of Earth and Environmental Sciences, The University of Queensland, St Lucia, QLD 4067 Australia; 2 School of Mathematics and Physics, The University of Queensland, St Lucia, QLD 4067 Australia; 3 Commonwealth Scientific and Industrial Research Organization (CSIRO) Oceans and Atmosphere, Queensland Biosciences Precinct (QBP), St Lucia, QLD 4067 Australia; 4 Centre for Marine Science and Innovation (CMSI), The University of New South Wales, Sydney, NSW 2052 Australia; 5 Sydney Institute of Marine Science, Mosman, NSW 2088 Australia; 6 Systems Ecology and Resource Management, Department of Organism Biology, Faculté des Sciences, Université Libre de Bruxelles - ULB, Avenue F.D. Roosevelt 50, CPi 264/1, 1050 Brussels, Belgium; 7 Ecology and Biodiversity, Laboratory of Plant Biology and Nature Management, Biology Department, Vrije Universiteit Brussel - VUB, Pleinlaan 2, VUB-APNA-WE, 1050 Brussels, Belgium

**Keywords:** Marine biology, Conservation biology, Marine biology, Conservation biology

Correction to: *Scientific Data* 10.1038/s41597-022-01528-7, published online 19 July 2022

The original version of this work reported that the Catch per unit effort (CPUE) categories had the same CPUE range for each species. This was detailed in the following sentence in the Data Records section:

Abundance refers to the categorical numeric levels of CPUE: 0 = no catch; 1 = 0.0–0.5 larvae·1,000 m^−3^; 2 = 0.5–1.0 larvae·1,000 m^−3^; 3 = 1.0–5.0 larvae·1,000 m^−3^; and 4 = > 5.0 larvae·1,000 m^−3^.

This was incorrect due to the CPUE definition varying according to species. While this does not affect the assignment of each species’ CPUE categories in the dataset (the numerical levels are unchanged), we have updated the above definition in the paper to explain the differences in CPUE ranges across species. The numeric levels have been removed from the sentence and replaced with a table of CPUE ranges for each species. This has been updated in the HTML and pdf versions of the paper, with the new table designated Table 2. The caption of figure 3 has also been updated to reflect this change, alongside an updated Figure 3 that provides CPUE numerical categories instead of the CPUE bins that are different for the different species.

