# Supplementary material for: A global, historical database of tuna, billfish, and saury larval distributions
Source: Sci Data. 2022 Jul 19;9:423. doi: 10.1038/s41597-022-01528-7 (PMC9296635; doi:10.1038/s41597-022-01528-7)
Supplement: Supplementary file 1 — Supplementary Figure S1 [file 41597_2022_1528_MOESM1_ESM.pdf]

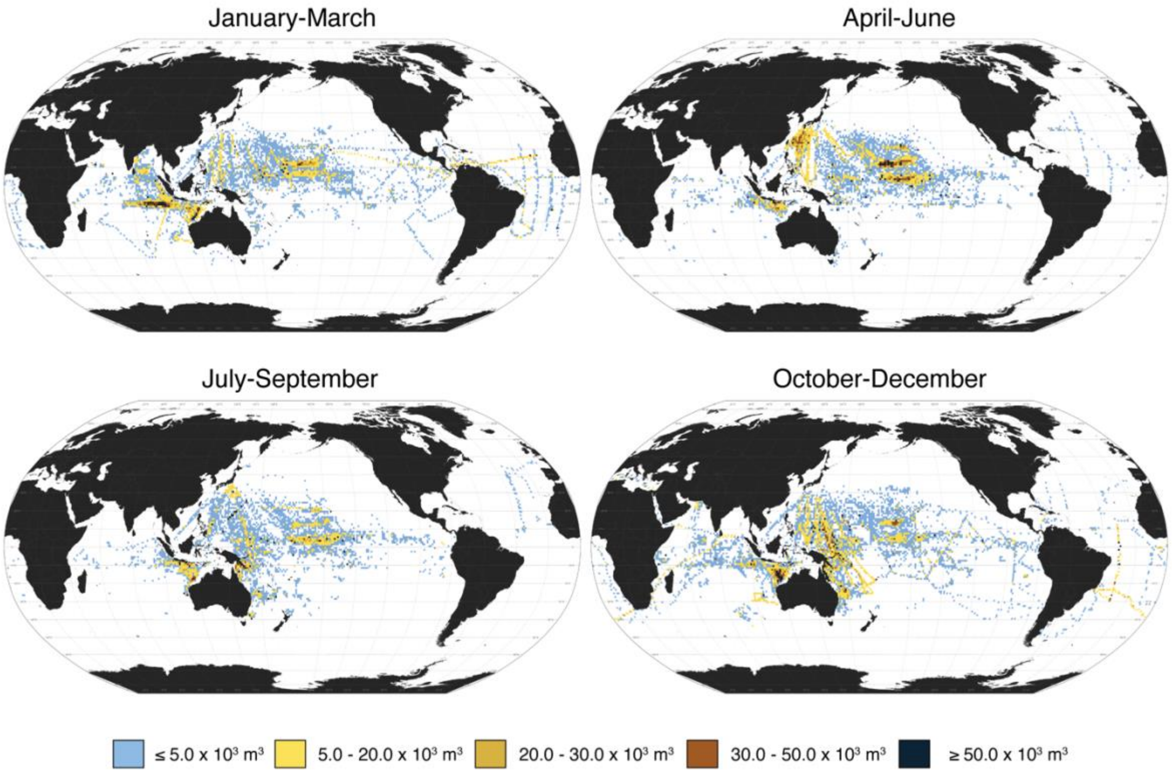

Figure S1. Volume of strained water. Seasonal 1°x1° maps of towing effort in terms of volume of strained water (expressed in x 10^3 m^3).
